# Identifying Plant Part Composition of Forest Logging Residue Using Infrared Spectral Data and Linear Discriminant Analysis

**DOI:** 10.3390/s16091375

**Published:** 2016-08-27

**Authors:** Gifty E. Acquah, Brian K. Via, Nedret Billor, Oladiran O. Fasina, Lori G. Eckhardt

**Affiliations:** 1 Forest Products Development Center, School of Forestry and Wildlife Sciences, Auburn University, 520 Devall Drive, Auburn, AL 36849, USA; bkv0003@auburn.edu; 2 Department of Mathematics and Statistics, Auburn University, Auburn, AL 36849, USA; billone@auburn.edu; 3 Center for Bioenergy and Bioproducts, Department of Biosystems Engineering, Auburn University, 350 Mell Street, Auburn, AL 36849, USA; fasinoo@auburn.edu; 4 Forest Health Dynamics Laboratory, School of Forestry and Wildlife Sciences, Auburn University, 602 Duncan Drive, Auburn, AL 36849, USA; eckhalg@auburn.edu

**Keywords:** process optimization, bioeconomy, forest biomass, fourier transform infrared spectroscopy, near infrared spectroscopy, linear discriminant analysis, principal component analysis

## Abstract

As new markets, technologies and economies evolve in the low carbon bioeconomy, forest logging residue, a largely untapped renewable resource will play a vital role. The feedstock can however be variable depending on plant species and plant part component. This heterogeneity can influence the physical, chemical and thermochemical properties of the material, and thus the final yield and quality of products. Although it is challenging to control compositional variability of a batch of feedstock, it is feasible to monitor this heterogeneity and make the necessary changes in process parameters. Such a system will be a first step towards optimization, quality assurance and cost-effectiveness of processes in the emerging biofuel/chemical industry. The objective of this study was therefore to qualitatively classify forest logging residue made up of different plant parts using both near infrared spectroscopy (NIRS) and Fourier transform infrared spectroscopy (FTIRS) together with linear discriminant analysis (LDA). Forest logging residue harvested from several *Pinus taeda* (loblolly pine) plantations in Alabama, USA, were classified into three plant part components: clean wood, wood and bark and slash (i.e., limbs and foliage). Five-fold cross-validated linear discriminant functions had classification accuracies of over 96% for both NIRS and FTIRS based models. An extra factor/principal component (PC) was however needed to achieve this in FTIRS modeling. Analysis of factor loadings of both NIR and FTIR spectra showed that, the statistically different amount of cellulose in the three plant part components of logging residue contributed to their initial separation. This study demonstrated that NIR or FTIR spectroscopy coupled with PCA and LDA has the potential to be used as a high throughput tool in classifying the plant part makeup of a batch of forest logging residue feedstock. Thus, NIR/FTIR could be employed as a tool to rapidly probe/monitor the variability of forest biomass so that the appropriate online adjustments to parameters can be made in time to ensure process optimization and product quality.

## 1. Introduction

Lignocellulosic biomass is a renewable and largely untapped source of feedstock that can be converted into biopower, liquid and gas fuels, and other biobased products via thermochemical and biochemical conversion pathways. The development of economically and environmentally sustainable sources of biomass can help countries to reduce their dependence of imported fossil fuels and diversify their energy portfolios.

Bioenergy accounts for 4% of the total primary energy consumption in the USA. The country utilizes approximately 200 million dry tons of biomass that is mostly sourced from forestlands for energy. In addition to this volume, some 93 million dry tons of forest biomass that is made up of 68 million dry tons of logging residue and 25 million dry tons of other removal residue, is, however, currently left onsite annually. It is estimated that the USA has the potential to harvest between 239 to 251 million dry tons of forest biomass on an environmentally and economically sustainable basis by 2030 as new markets and technologies emerge [1,2].

Forest logging residue is mostly made up of tops, branches and limbs of merchantable trees, salvageable dead trees and small unmerchantable trees. Owing to the different plant part components, there is variability in the composition and quality of this resource. The heterogeneity of forest logging residue can influence important properties that dictate feedstock quality for specific applications. These physical, chemical and compositional characteristics can either setback or boost biomass conversion processes. A good understanding of these properties is crucial in the establishment of a successful biomass conversion facility. Furthermore, an ability to monitor the properties of the feedstock entering the process and accordingly making the necessary online adjustments in process parameters will be essential in the optimization, quality assurance and cost-effectiveness of conversion technologies in the emerging biofuel/chemical industry. A first step in this direction will be a system that can probe the variability in the composition of a batch of feedstock. Such a system should work in processing/online conditions, provide rapid and accurate analysis of a large number of heterogeneous samples in a non-destructive manner, be easy to use and be cost-effective. Infrared spectroscopy has been shown to have the potential for use in process optimization applications.

Infrared spectroscopy is the measurement of the absorption, transmittance or reflectance of infrared light by a sample. The infrared region is the wavelength range of 780 nm–1 mm (i.e., wave number range of 12,820 cm^−1^–10 cm^−1^) that lies between the visible and microwaves regions of the electromagnetic spectrum. The region is subdivided into near infrared (NIR), mid infrared (MIR) and far infrared (FIR). NIR region lies between the wavelength range of 780 nm to 2500 nm (i.e., 12,820 cm^−1^ to 4000 cm^−1^); and MIR from 2500 nm to 5600 nm (i.e., 4000 cm^−1^ to 785 cm^−1^) [3,4,5]. Near infrared spectroscopy (NIRS) uses near infrared light to detect overtones and combinational vibrations of the molecular constituents of the material under study; whereas Fourier transform infrared spectroscopy (FTIRS) uses mid infrared light to detect primarily functional and fundamental vibrations. Absorption bands that commonly occur in the NIR region and MIR region are overtones and combinations of fundamental vibrations of O-H, C=O, N-H, -COOH, C-H, aromatic C-H groups and S-H functional groups; and can, thus, give the chemical and physical properties of a material [6,7,8].

NIRS and FTIRS have mostly been used for quantitative analysis in the forest products industry and emerging bioeconomy [9,10,11,12,13,14,15,16,17,18,19,20,21,22,23,24,25,26]. In an earlier study, we employed NIRS in the prediction of some important properties of forest logging residue for bioenergy, fuel and chemical applications [9]. It has also been used in the quantitative prediction of cellulose, cellulose crystallinity, hemicellulose, lignin and extractives in wood and lignocellulosic biomass [10,11,12,13,14]. In addition, NIRS has successfully been used to model secondary properties that correlate well with wood chemistry including density [15], compression wood [16], tracheid morphology [17], mechanical properties [18], kraft pulp yield [19] and energy content [20]. Although NIRS does not interact directly with inorganic species, some recent studies have been able to predict the ash content of wood and biomass with varying degree of success [21,22].

Just like NIRS, FTIRS has been used to predict the density of loblolly pine [23]. Models were also built for the higher heat value (HHV), volatile matter, fixed carbon and ash content of torrefied biomass using ATR-FTIR spectra [24]. Nuopponen et al. (2006) [25] used DRIFT-FTIR spectra to model lignin, cellulose, extractives and density using fifty clones of sitka spruce, twenty-four Ghanaian hardwoods and twenty scots pine. In an earlier study, Tucker et al. (2000) [26] used partial least squares models developed with FTIR spectra to quantify the glucose, mannose, galactose, xylose, acetic acid and 5-hydroxymethyl-2-furfural (HMF) of dilute acid pretreated biomass.

With respect to the utilization of infrared spectroscopy for qualitative analysis, different wood species were separated using principal component analysis (PCA) and partial least squares discriminant analysis (PLS-DA) on NIR spectra [27]. The researchers were however not as successful in their attempt to distinguish between wood samples from different locations. Other studies classified wood thermally treated under different conditions [28], herbaceous biomass [29], botanical fractions of cornstover [30] and wood-based materials [31] using NIRS coupled with chemometric methods such as soft independent modeling of class analogies (SIMCA), Mahalanobis’ generalized distance, Kernel PLS and PCA among others. Similarly, FTIRS has been used in the discrimination and classification wood and wood-based materials [32,33,34,35].

It is hypothesized that, since infrared light is sensitive to the chemical composition of a sample, NIR and/or FTIR spectra can be used to separate out materials that have different chemistry. The objective of this study was, therefore, to use both near infrared spectroscopy (NIRS) and Fourier transform infrared spectroscopy (FTIRS) together with PCA and linear discriminant analysis (LDA) in the qualitative classification of *Pinus taeda* (loblolly pine) forest logging residue made up of different plant parts in a comparative study. As mentioned earlier, forest logging residue is a largely untapped resource that can play a key role in the bioeconomy as technologies advance in biomass supply chain logistics and new markets emerge for biofuel and other bioproducts.

## 2. Experimental Section

### 2.1. Materials

Forest biomass was obtained during harvesting operations on loblolly pine plantations located on several forest tracts in Greenville, Alabama, (31°49′52.583′′ N, 86°37′39.241′′ W) and Georgiana, Alabama (31°38′24.313′′ N, 86°44′21.991′′ W). The stands were between 10 and 18 years old, and the diameter at breast height (DBH) of trees ranged from 10 to 20 cm. Biomass was made up of ‘Clean wood’, ‘Slash’ and ‘Wood & bark’. Clean wood was sampled from either debarked disks that were removed at 5 feet interval along the main stem or from the whole debarked stems of loblolly pine trees. All disks from a tree were combined into a single sample. Slash material is the limbs and foliage of delimbed loblolly pine trees. For ‘Wood and bark’, material was sampled from the wood and bark of southern pines (mostly loblolly pine) whole stems. Except for the debarked disks that were transported and chipped at Auburn University, AL, USA, all other materials were sampled onsite from chip streams at chipper discharge. A sampling pipe was raised into a chip stream 8–10 times per load. Final representative subsamples were obtained in the lab through coning and quartering. Harvesting, chipping and sampling of biomass spanned several months; from November 2010 to March 2012.

Material used in this study is representative of biomass feedstock that will most likely be used in a bioprocessing plant located in this region. It is typical of feedstock material a manufacturing facility will be acquiring either as pre-commercial thinnings, whole tree utilization of loblolly pine dedicated as an energy crop, or pulpwood chips.

### 2.2. Methods

#### 2.2.1. Determination of Chemical Composition and Ash Content

The major chemical components of biomass, i.e., cellulose, hemicellulose, lignin and extractives, were measured via conventional wet chemistry and High Performance Liquid Chromatography (HPLC) (Shimadzu Corporation, Kyoto, Japan). Samples were prepared for analysis by grinding through a 40-mesh screen using a Wiley Mill (Model 3383-L10, Thomas Scientific, Swedesboro, USA). 

Extractive content of forest logging residue was determined following NREL/TP-510-42619 and TAPPI T204. Test samples were extracted in 150 mL of industrial grade acetone for 6 h in a Soxhlet Apparatus. The amounts of carbohydrates and total lignin were determined as described in NREL/TP-510-42618. After a two-step acid hydrolysis of extractive-free samples, HPLC was employed in the measurement of monomeric sugars (i.e., glucose, xylose, galactose, arabinose and mannose). The sum of all monomeric sugars gave the holocellulose content. Cellulose was computed as $\text{glucose}-\left(\frac{1}{3}\;\times \;\text{mannose}\right)$ and hemicelluloses computed as the difference between holocellulose and cellulose. The total lignin was calculated as the sum of acid soluble lignin (ASL) and acid insoluble lignin (AIL). ASL was determined with a UV-Visible spectrophotometer immediately following hydrolysis. Absorbance of a test sample was measured at the recommended wavelength of 240 nm, ensuring that it ranged between 0.7 and 1.0. The ash content of forest logging residue was determined as residue after dry oxidation of test samples at 575 °C, as specified in NREL/TP-510-42622.

For each plant part/group, the chemical composition and ash content was determined using ten of the seventeen samples (i.e., n = 10). Experiments were run in duplicate for each sample. Knowledge of the chemical composition and ash content of the different plant parts will be useful in the interpretation and elucidation of PC and discriminant analysis.

#### 2.2.2. Infrared Spectra Collection

Spectra of forest biomass were acquired with a PerkinElmer Spectrum 400 FT-IR/FT-NIR Spectrometer. The FT-IR unit was equipped with a diamond crystal attenuated total reflectance device (i.e., ATR-FTIR) and a torque knob to ensure consistent application of pressure to samples during spectra collection. Samples were ground to pass an 80-mesh screen and oven dried for 4 h before spectra acquisition. Spectra were collected at 1 cm^−1^ interval from 10,000 cm^−1^ to 4000 cm^−1^ for near infrared and from 4000 cm^−1^ to 650 cm^−1^ for mid infrared. This resulted in 6000 and 3500 data points/variables for NIRS and FTIRS respectively. A sample was scanned thirty-two times at a resolution of 4 cm^−1^ and averaged into one spectrum for analysis. For NIRS, spectrum of a Spectralon was taken as the background reference sample every 10 min to correct for potential drifts with time. In the case of FTIRS, the background was spectrum of a clear window. Due to the very high dimension of these data sets, spectra were compressed to 10 cm^−1^ interval before exporting to SAS 9.4 for further analysis. An earlier study by Via et al. (2011) [23] showed that such compressing/averaging allows the analysis of large data matrices without compromising the integrity of results.

#### 2.2.3. Multivariate Data Analysis

##### Principal Component Analysis (PCA)

PCA is a widely used statistical technique which attempts to explain the covariance structure of data by using a small number of components. These components are linear combinations of the original variables, and often allow for an interpretation and a better understanding of the different sources of variation. PCA is concerned with data reduction. Therefore, it is commonly used for the analysis of high-dimensional data which arise frequently in chemometrics, computer vision, engineering, genetics and other fields. PCA is, thus, used as a preliminary step of data analysis, followed by further multivariate statistical methods.

As an initial step, PCA was employed to reduce dimension of the data (p = 600 wavelengths for NIR spectra and p = 335 for FTIR spectra). PCA takes a set of correlated variables (as is the case in IR spectra) and transforms them into a smaller set of uncorrelated variables known as principal components (PCs) while maintaining as much of the information in the original data as possible. In other words, assuming that there are n observations X_ij_ on p correlated variables X_1_, X_2_, …, X_p_, i = 1, …, n, j = 1, …, p, PCA finds new uncorrelated Z_1_, Z_2_,…,Z_p_ that are linear combinations of X_1_, X_2_, …,X_p_ as

Z_i_ = e_i1_X_1i_ + e_i2_X_2i_ + ……+ e_ip_X_pi_ & Var(Z_i_) = λ_i_, i = 1, …, p

where λ_i_s (λ_1_ > λ_2_ > … > λ_p_) and e_i_ are the eigenvalues and the corresponding eigenvectors of the covariance matrix of data matrix X (n by p). The coefficient, e_ij_ is a measure of the importance of the jth original variable to the ith PC irrespective of the other variables. The coefficients, known as component loadings or eigenvectors are proportional to the correlation between Zs and Xs and can be used in interpreting PCs. The values of the ith principal component are called the PC scores.

The first PC (i.e., Z_1_) corresponds to the direction in which the projected observations have the largest variance (i.e., Var(Z_1_) = λ_1_, which is the largest eigenvalue). The second component is then orthogonal to the first and again maximizes the variance of the data points projected on it. Continuing in this way produces all the principal components, which correspond to the eigenvectors of the covariance matrix of the data matrix X. In order to determine the number of components, the Proportion of Explained Variance (>99.5%) was used.

For model calibration and validation, a 5-fold cross validation was utilized due to the relatively small sample size (i.e., n = 51). The data set was randomly split into five blocks prior to PCA. Then, PCA was performed on standardized variables by using the correlation matrix of raw NIR and FTIR spectra by employing the PRINCOMP procedure in SAS. Four blocks were used together at a time as the training data for calibration and the remaining one block as the test data for validation. This was repeated until each of the five blocks was used as an independent test data (i.e., five total runs). As such, for each run, the data used for validation was independent/exclusive of the data used in developing the classification function. For each run, component loadings of the training data set were used to score the test data set.

##### Linear Discriminant Analysis (LDA)

Scores of retained PCs were used as input data for linear discriminant analysis. LDA is a supervised pattern recognition technique that seeks to find one or several linear functions or discriminants of the dependent variables that can be used to separate out classes/groups. Groups to which observations belong to are known and are defined by the multivariate data structure of its observations. LDA uses these structures to establish rules that allow new unknown samples to be assigned to one or another class [36,37]. Before classification, there is the natural probability (i.e., prior probabilities) that samples belong to one of the labeled groups and after classification there is also a probability (i.e., posterior probabilities) that samples belong to a group. The difference in prior and posterior probabilities enables the allocation of objects to one of the groups. Performances of discriminant functions were evaluated by their error rates or misclassification probabilities. The DISCRIM Procedure in SAS was used for LDA.

## 3. Results and Discussion

**Figure 1:**
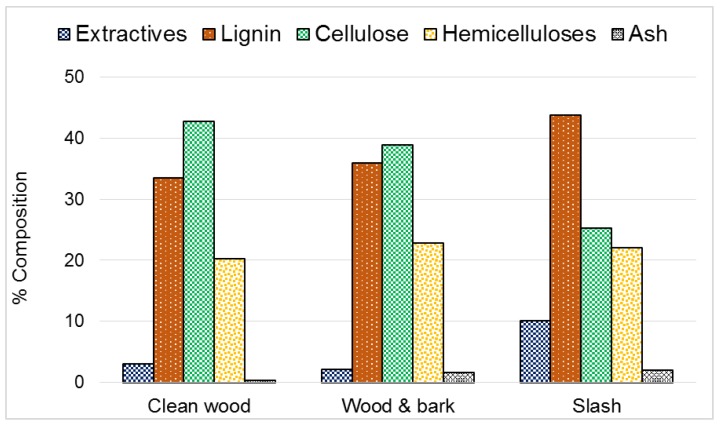
Chemical composition and ash content of forest logging residue.

The major chemical composition and ash content as determined in the three plant part components of forest logging residue is presented in Figure 1. There were statistical differences (significance level of 0.05) between the plant parts for all properties measured. Clean wood and Slash were the most different, while the chemical make-up of Wood and bark was generally more like Clean wood. For instance, Slash had the highest amount of lignin (44%), with Wood and bark and Clean wood having 36% and 34% respectively. Additionally, the 2% ash in Slash was statistically higher than the 1.6% in Wood and bark and 0.4% in Clean wood.

### 3.1. Infrared Spectra

**Figure 2:**
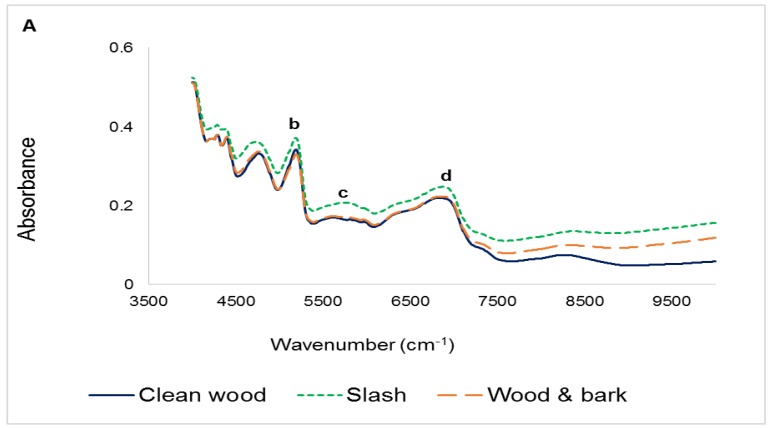

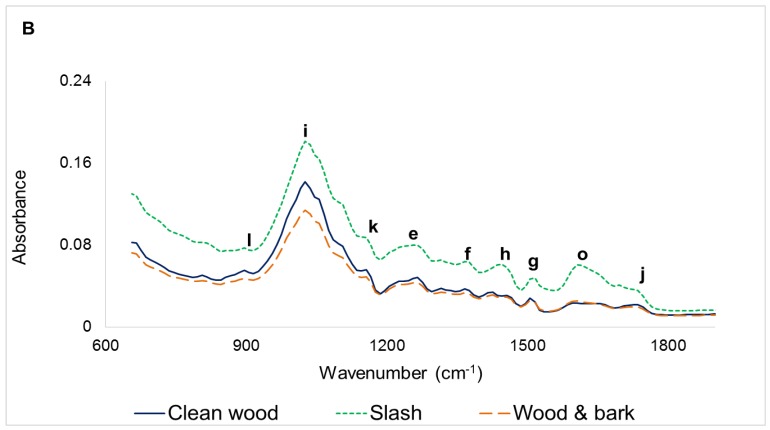
Raw NIR (**A**) and FTIR (**B**) spectra of the different types of forest logging residue.

Averaged NIR and FTIR spectra of the three plant part components of forest logging residue used in this study are presented in Figure 2. There was a general trend in the absorbance of near infrared and mid infrared by the plant parts. There were however variations in the intensity of light absorbed. For both NIR and FTIR, Slash absorbed the most. Clean wood absorbed the lowest amount of energy for a good portion of the near infrared region, but in the mid infrared region, its absorbance values were slightly higher or lower than the values for Wood and bark. Large baseline shifts noted in the 7100 to 10,000 cm^−1^ region might have resulted from the different ash contents of the three biomass types, Figure 1 [38]. Although infrared light interacts directly with only organic compounds in materials, these interactions may be influenced by the presence of their associated inorganic species [39].

In the near infrared region (Figure 2A), the absorbance peaks occurring from 4000 cm^−1^ to 5000 cm^−1^ are as a result of the interactions of O-H, C-H and N-H functional groups interacting with one another (i.e., combination bands). Peaks have also been ascribed to specific chemical constituents of lignocellulosic biomass: (a) 4765 cm^−1^ results from O-H and C-H stretching and deformation vibration of cellulose (and xylan); (b) 5205 cm^−1^ is due to the asymmetric stretching and/or deformation of O-H in water; and (c) 5845 cm^−1^ credited to the first overtone stretching of C-H in hemicelluloses. In addition, the peak at (d) 6875 cm^−1^ has been attributed to the first overtone of O-H stretching of phenolic groups in lignin [14,40].

As in the near infrared region, peaks arise in the mid infrared region due to the presence of functional groups in biomass. Although this region ranges from 4000 to 600 cm^−1^, the fingerprint region (1800 to 600 cm^−1^) is usually used for analysis because it contains the most spectral information pertaining to the molecular/chemical composition of a material (Figure 2B). According to the literature, bands at (e) 1270 cm^−1^; (f) 1365 cm^−1^; (g) 1505 cm^−1^ and (h) 1435 cm^−1^ have been associated with lignin; (e) and (f) result from guaiacyl ring breathing and syringyl ring breathing respectively, whereas (g) is due to the aromatic skeletal vibration with C=O stretch. For the carbohydrates, C=O stretch of unconjugated ketones mostly in hemicellulose generate bands at (i) 1025 cm^−1^ and (j) 1735 cm^−1^; whereas the peaks at (k) 1154 cm^−1^ and (l) 895 cm^−1^ result respectively from C-O-C stretching and P-chains of cellulose. Furthermore, the peak at (m) 2935 cm^−1^ outside the fingerprint range (Figure 2B inset) have been associated with the bending and stretching of C-H, as well as its aromatic ring vibration in lignin, while that occurring at (n) 3345 cm^−1^ is due to N-H stretching. Spectra of Slash had a very prominent peak at (o) 1635 cm^−1^ compared to Clean wood and Wood and bark. This has been attributed to C-O stretching of conjugated or aromatic ketones and/or C=O stretching vibration in flavones [22,25,33,35].

### 3.2. Principal Component Analysis

Partial results from PC analysis showing the first ten PCs is presented in Table 1. A preset criteria for the number of PCs to include in further analysis was that the eigenvalue of a PC should be more than 0.7 (i.e., PCA on the correlation matrix) and the cumulative variance should be greater or equal to 99.5%. In addition, the Scree Test Criterion was used. A Scree diagrams plots λi against i for i = 1, …, q; and λ is the eigenvalues. The point at which the curve begins to straighten out indicates a cut-off point. Based on Table 1 and the Scree plots, the first six PCs were tentatively retained for linear discriminant analysis.

The first six PCs out of the possible 600 for NIRS and 335 for FTIRS were able to account for over 99.5% of the total variation in the data. For NIRS, PC 1 and PC 2 accounted for 76% and 16%, respectively, of the spectra data; in the case of FTIRS, they were 81% and 15%, respectively.

**Figure 3:**
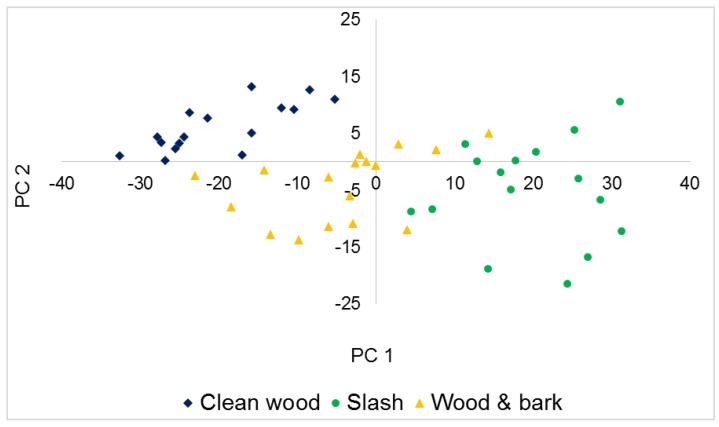
NIR scores plot of PC 1 versus PC 2.

Employing PCA as a preliminary classification tool, scores of the retained PCs were plotted against each other. In Figure 3, a graph of the scores of raw near infrared spectra for PC 1 and PC 2 is presented. Separation was better along PC 1; Clean wood clustered furthermost from Slash, with Wood and bark in between the two classes.

**Figure 4:**
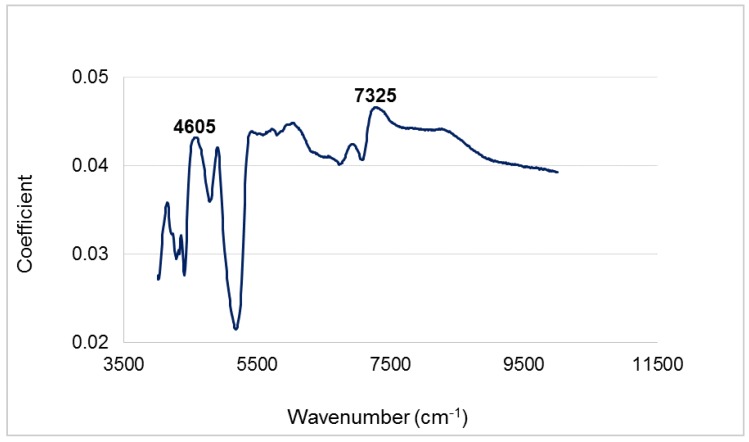
NIR loadings plot of PC 1 showing significant peaks.

According to the loading plot of PC 1 (Figure 4), cellulose content (peaks at 4605 and 7325 cm^−1^) was a good initial separator of the different plant parts as it had higher coefficient values. This could be backed by results from the conventional chemical analysis. Reviewing Figure 1, Clean wood had the highest percentage of cellulose (43%), followed by Wood and bark (39%), then Slash (25.2%). Thus, on the PC 1 axis, the three biomass types separated from left to right due to decreasing cellulose content. Other significant coefficients noted in NIR spectra loadings that contributed to the classification of the three groups of forest logging residue were at 7095 cm^−1^ in PC 3, which is attributed to the phenolic groups in lignin and/or extractives and a peak at 5835 cm^−1^ in PC 4 occurring due to C-H stretching in hemicelluloses. Again, results from PC analysis were buttressed and elucidated by chemical composition determined via conventional laboratory methods.

**Figure 5:**
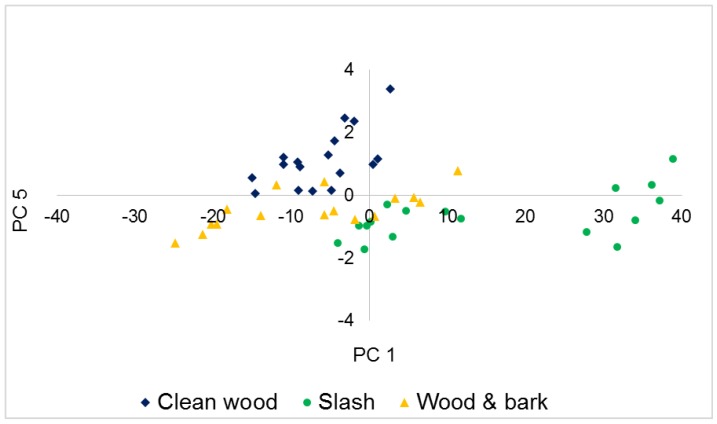
FTIR scores plot of PC 1 versus PC 5.

**Figure 6:**
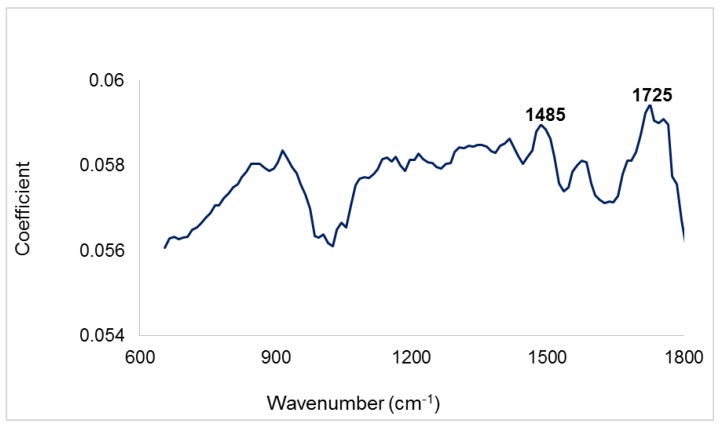
FTIR loadings plot of PC 1 showing significant peaks.

In the case of FTIRS, a plot of PC1 against PC5 (Figure 5) gave the best initial separation with better separation along PC 1. This was however not as distinct especially between Clean wood and Wood and bark as was seen in the scores plot of NIR spectra. A characteristic cellulose peak occurring at 1725 cm^−1^ was again observed in the loadings of PC 1, Figure 6. In addition, the large loadings coefficients of 1485 cm^−1^ suggests that vibrations attributed to both lignin and polysaccharides also contributed to the initial distinction of different biomass types in the mid infrared region.

### 3.3. Linear Discriminant Analysis (LDA)

**Figure 7:**
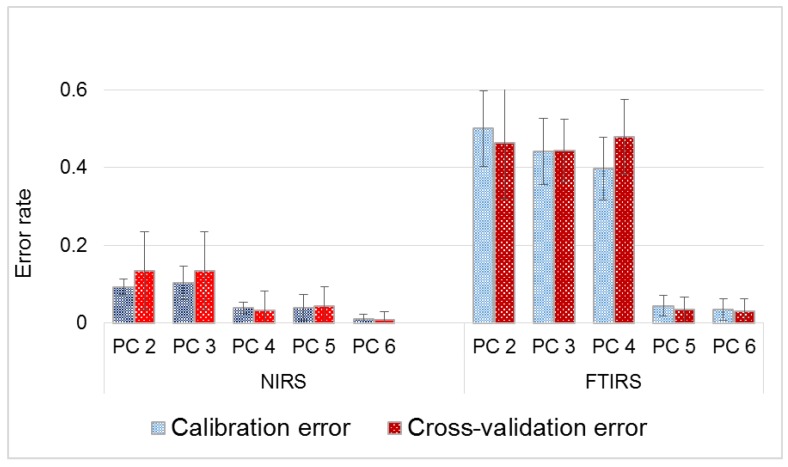
Effect of changing number of PCs on classification error.

The first six PCs retained (chosen based on the eigenvalue, variance explained and Scree Test Criteria) were used in LDA. Examining the effect the inclusion of PCs had on errors associated with classification (Figure 7), the discriminant functions (Table 2) developed with four and five PCs were chosen as the optimum for NIRS and FTIRS, respectively. These selections were made because the difference in errors for the training data set and test data were the least. Furthermore, standard deviation of the five folds used in model calibration were smallest for the selected number of PCs.

Calibration errors were computed using the Lachenbruch’s Holdout procedure, whereby all samples except the first sample were used in building the discrimination function to classify the first, then the second is held out and the process repeated until all samples have been used as single-element test sets in a fold [41]. Unlike errors generated in five-fold cross-validation of the test set, errors estimates by the Lachenbruch’s Holdout procedure (i.e., a leave-one-out cross-validation technique) may be overoptimistic due to the exclusivity of the one-sample test data. Up until inclusion of the fifth PC, errors associated with FTIRS-based discriminant functions were very high. For instance, while the model built from NIR spectra using four PCs had only 4% and 3% as misclassification errors of cross-validation for the respective training data and test data, errors for FTIRS were 40% and 48% respectively. Studies that have been conducted to compare NIRS and FTIRS in the quantitative or qualitative analysis of lignocellulosic biomass and plant-based materials have reported differing results mostly in favor of the latter, albeit slightly [24,42,43,44,45]. Better performance of FTIRS relative to NIRS have been attributed to the fundamental vibrations in the MIR region as opposed to overlapping and weaker overtone and combination bands observed in the NIR region. The abundance of absorption bands especially in the fingerprint region of the former make identification/ qualification of molecular structures easier. On the other hand, chemometric techniques are usually required in order to extract relevant information in the latter [6].

The generalized squared distance (Table 3) gives an indication of the degree of separation between classes in space. A new/unknown sample is classified into a group if it is similar enough to the other members, otherwise it is rejected. According to Table 4, Slash was most distinct from Clean wood and less so from Wood and bark. These results are in agreement with that from PCA, as can be seen in Figure 3 and Figure 5 when PC scores were plotted.

From the error count estimate in Table 4, the performance of developed functions in predicting the class of independent test samples were computed.

As seen in Table 5, linear discriminant functions developed with NIR and FTIR spectra were able to classify the plant part components of logging residue with over 96% overall accuracy. Clean wood was the easiest to identify, while Wood and bark generally had the highest misclassification rate. This was to be expected considering the plant part makeup of the three materials studied. Moreover, from the chemical and ash content analysis, it was determined that the properties of Wood and bark were more similar to the other two plant part components.

### 3.4. Remarks

Ideally, samples used in model validation should be independent of the training dataset. However, this cannot always be the case due to limited resources. When sample size is small, researchers have employed cross validation (CV) to test the performance of calibration models instead of splitting up the data into a single training set and test set. A commonly used technique is the leave-one-out CV method. In this procedure, n − 1 samples are used in training a model that is validated with the held out sample. This is repeated n times until each observation has been used as validation data. The advantage of this approach is that, it uses the maximum available data in both model training and validation. However, due to the exclusivity of the one-sample test data, the errors estimates may be overoptimistic. To overcome this potential problem, the current study opted for a five-fold cross validation. This ensured that a test dataset comprised of observations with varying backgrounds—for instance, different age, DBH or site. Additionally, taking the average of five repetitions instead of just one experimentation gives a significantly better estimate of the errors.

Another strength of the developed classifier lies in the range of samples used. Materials used in this study are representative of biomass feedstock that will most likely be used in a bioprocessing plant located in this region. Loblolly pine (and southern pine on one site) that were 10 to 18 years old, with a DBH range of 10–20 cm from several forest sites were used. This is typical of feedstock material a manufacturing facility will be getting either from pre-commercial thinnings, loblolly pine dedicated as an energy crop or pulpwood chips. Thus, models constructed in this study are robust and will perform well in classifying similar feedstock in this region.

The aim of this study was to demonstrate that NIR and FTIR can be used to rapidly identify what a batch of feedstock is made up of, as this, as is known will influence the chemical composition. A traditional way to do this is probably by visual inspection. Compared to this, NIR/FTIR has a higher throughput, and will have fewer errors, especially for comminuted feedstock. With this information, on-time adjustments could be made in the process parameters so that product yield and quality can be optimized/assured. Such information could also be used in future feedstock acquisition.

Any processing plant employing NIR/FTIR as a classification tool will first have to calibrate their system with samples that is within the range of materials characteristic to their locality. Apart from this qualitative probing/monitoring, a facility’s system could also be trained to provide quantitative information, such as the cellulose, lignin, ash or energy content of feedstock coming into the process.

## 4. Conclusions

This study demonstrated that NIR or FTIR spectroscopy coupled with PCA and LDA has the potential to be used as a high throughput tool in classifying the plant part makeup of a batch of forest logging residue feedstock. Peaks noted at 4605 and 7325 cm^−1^ (i.e., NIR) in the loading plot of PC 1 suggested that the significantly different amount of cellulose contributed to the initial separation of the different plant parts. In the mid infrared region (i.e., FTIRS) preliminary separation was made possible due to the varying concentrations of lignin and polysaccharides. Both NIRS and FTIRS based linear discriminant functions had very good classification accuracies (i.e., 96%) even though an extra variable/PC was needed to achieve this with FTIRS modeling.

Applications for this study include its use as a rapid tool to probe/monitor the variability of forest logging residue so that the appropriate online adjustments to parameters can be made in time to ensure process optimization and product quality.

## Figures and Tables

**Table 1:** Eigenvalues of the correlation matrix ^1^.

	NIRS			FTIRS		
PC	Eigenvalue	Proportion of Variance Explained (%)	Cumulative Variance (%)	Eigenvalue	Proportion of Variance Explained (%)	Cumulative Variance (%)
1	456.4 (4.8)	76.1	76.1	271.0 (5.2)	80.9	80.9
2	95.4 (3.7)	15.9	92.0	49.9 (4.0)	14.9	95.8
3	41.8 (6.5)	7.0	98.9	9.6 (3.6)	2.9	98.7
4	3.6 (1.3)	0.6	99.5	1.8 (0.3)	0.6	99.2
5	1.3 (0.1)	0.6	99.7	1.3 (0.2)	0.4	99.6
6	0.85 (0.08)	0.14	99.88	0.29 (0.04)	0.09	99.68
7	0.36 (0.03)	0.06	99.94	0.16 (0.03)	0.05	99.73
8	0.13 (0.01)	0.02	99.96	0.09 (0.01)	0.03	99.76
9	0.06 (0.01)	0.01	99.97	0.07 (0.01)	0.02	99.78
10	0.04 (0.01)	0.01	99.98	0.06 (0.01)	0.02	99.80

^1^ Values are the means of the five folds used as training data sets. SD values in brackets.

**Table 2:** Linear discriminant functions used for classifying plant part components.

	NIRS			FTIRS		
Variable	Clean Wood	Slash	Wood and Bark	Clean Wood	Slash	Wood and Bark
Constant	−6.13 (0.71)	−9.29 (3.51)	−2.14 (0.36)	−6.89 (1.73)	−10.34 (3.96)	−2.02 (0.51)
PC1	−0.39 (0.08)	0.51 (0.17)	−0.11 (0.04)	−0.4 (0.08)	0.56 (0.16)	−0.14 (0.03)
PC2	0.42 (0.03)	−0.43 (0.04)	0.02 (0.06)	−0.16 (0.06)	0.2 (0.07)	−0.05 (0.04)
PC3	−0.04 (0.06)	0.07 (0.05)	−0.03 (0.01)	0.21 (0.23)	−0.41 (0.35)	0.22 (0.13)
PC4	1.23 (0.35)	−2.35 (0.66)	1.07 (0.19)	−3.06 (1.74)	3.94 (1.52)	−1.12 (0.39)
PC5	-	-	-	6.56 (1.66)	−7.59 (2.84)	0.78 (0.41)

**Table 3:** Generalized squared distances of the three plant part components of forest logging residue.

	NIRS			FTIRS		
From/Into	Clean Wood	Slash	Wood and Bark	Clean Wood	Slash	Wood and Bark
Clean wood	2.2 (0.3)	52.5 (12.9)	10.6 (1.6)	2.2 (0.3)	59.9 (17.8)	11.1 (1.7)
Slash	52.5 (12.9)	2.2 (0.3)	28.2 (8.2)	59.9 (17.8)	2.2 (0.3)	30.2 (7.3)
Wood & bark	10.6 (1.6)	28.2 (8.2)	2.2 (0.3)	11.1 (1.7)	30.3 (7.3)	2.2 (0.3)

**Table 4:** Five-fold cross-validation summary of error count estimates (%) for plant part component ^1^.

	NIRS				FTIRS			
	Clean Wood	Slash	Wood and Bark	Total	Clean Wood	Slash	Wood and Bark	Total
Rate	0%	3.3%	6.7%	3.2%	0%	3.3%	8.3%	3.4%

^1^ Values are averages of the five groups of test samples used in validation.

**Table 5:** Classification rates for forest logging residue ^1^.

	NIRS					FTIRS				
	Plant Part Component									
Test Sample	Clean Wood	Slash	Wood & Bark	Total	% Correct Classification	Clean Wood	Slash	Wood & Bark	Total	% Correct Classification
Clean Wood	17	0	0	17	100	17	0	0	17	100
Slash	0	16	1	17	96.7	0	16	1	17	96.7
Wood and bark	1	0	16	17	93.3	2	0	15	17	91.7
% Total Accuracy					96.7 (3.3)					96.1 (4.2)

^1^ Calculated based on error count estimates (in Table 4). SD values in brackets.
